# Molybdenum limitation of microbial nitrogen assimilation in aquatic ecosystems and pure cultures

**DOI:** 10.3389/fmicb.2012.00331

**Published:** 2012-09-13

**Authors:** Jennifer B. Glass, Richard P. Axler, Sudeep Chandra, Charles R. Goldman

**Affiliations:** ^1^School of Earth and Space Exploration, Arizona State UniversityArizona, AZ, USA; ^2^Natural Resources Research Institute, University of Minnesota DuluthDuluth, MN, USA; ^3^Department of Natural Resources and Environmental Science, University of Nevada RenoReno, NV, USA; ^4^Department of Environmental Science and Policy, University of California DavisDavis, CA, USA

**Keywords:** molybdenum, nitrate reductase, nitrogen fixation, limnology, trace elements, microbes, nutrient limitation, enzyme activity

## Abstract

Molybdenum (Mo) is an essential micronutrient for biological assimilation of nitrogen gas and nitrate because it is present in the cofactors of nitrogenase and nitrate reductase enzymes. Although Mo is the most abundant transition metal in seawater (107 nM), it is present in low concentrations in most freshwaters, typically <20 nM. In 1960, it was discovered that primary productivity was limited by Mo scarcity (2–4 nM) in Castle Lake, a small, meso-oligotrophic lake in northern California. Follow up studies demonstrated that Mo also limited primary productivity in lakes in New Zealand, Alaska, and the Sierra Nevada. Research in the 1970s and 1980s showed that Mo limited primary productivity and nitrate uptake in Castle Lake only during periods of the growing season when nitrate concentrations were relatively high because ammonium assimilation does not require Mo. In the years since, research has shifted to investigate whether Mo limitation also occurs in marine and soil environments. Here we review studies of Mo limitation of nitrogen assimilation in natural microbial communities and pure cultures. We also summarize new data showing that the simultaneous addition of Mo and nitrate causes increased activity of proteins involved in nitrogen assimilation in the hypolimnion of Castle Lake when ammonium is scarce. Furthermore, we suggest that meter-scale Mo and oxygen depth profiles from Castle Lake are consistent with the hypothesis that nitrogen-fixing cyanobacteria in freshwater periphyton communities have higher Mo requirements than other microbial communities. Finally, we present topics for future research related to Mo bioavailability through time and with changing oxidation state.

## Introduction

Molybdenum (Mo) is an essential micronutrient in all three domains of life. Mo is particularly important for microbial nitrogen (N) assimilation due to its presence in nitrogenase, the enzyme that performs N_2_ fixation and in nitrate reductase, the enzyme that performs the first step in nitrate (NO^−^_3_) assimilation, reduction of NO^−^_3_ to nitrite (NO^−^_2_). For other biological functions of Mo, see Sigel and Sigel (2002). When ammonium (NH^+^_4_) is present over a certain threshold, it is either preferred over NO^−^_3_ or inhibits NO^−^_3_ uptake (Dortch, 1990), and Mo requirements decrease because NH^+^_4_ assimilation does not require Mo. While Mo is the most abundant transition metal in seawater (107 nM; Collier, 1985), Mo concentrations are low in most freshwaters (<20 nM), which raises the question of whether N assimilation in low-Mo freshwater ecosystems is limited by Mo availability when NH^+^_4_ concentrations are scarce enough that organisms must rely on N_2_ and NO^−^_3_ for their N sources.

In this paper, we review both laboratory and field experiments in aquatic (and briefly, soil) environments that have tested the effect of varying Mo concentration on N assimilation via N_2_ fixation and NO^−^_3_ reduction. This research dates back to the first half of the twentieth century, when studies with pure cultures showed that removal of Mo from growth media induced symptoms of N-limitation in N_2_-fixing bacteria (Bortels, 1930, 1940) and NO^−^_3_-assimilating fungi (Steinberg, 1937). Since then, it has been shown that N_2_ fixation requires more Mo than NO^−^_3_ assimilation, scarce Mo limits N assimilation in a wide range of natural environments, and Mo bioavailability may decrease with increasing atmospheric CO_2_, leading to suppressed terrestrial N_2_ fixation (Hungate et al., 2004). We focus particular attention on previous and current research of Mo limitation of N assimilation at Castle Lake, CA, the only aquatic habitat in the world that has been the subject of multiple decades of Mo limitation research. We summarize new results on the effect of Mo additions on total protein abundance and activity of enzymes involved in NO^−^_3_ assimilation in Castle Lake, as well as high-resolution Mo depth profiles that suggest Mo may be limiting N_2_ fixation by lake periphyton communities. Finally, we discuss future research directions and novel techniques that hold promise for advancing research on this topic.

## Pure culture studies

Research on Mo requirements for N assimilation in aquatic microbes has largely focused on heterocystous cyanobacteria for N_2_ fixation studies and green algae for NO^−^_3_ assimilation studies (Table 1). In instances where growth of heterocystous cyanobacteria was compared on different N sources, Mo requirements were consistently higher in the N_2_-fixing than NO^−^_3_-grown condition (Table 1), as predicted based on specific activity of the nitrogenase vs. nitrate reductase enzymes (see below).

**Table 1:** **Previous studies of Mo dependence of N_2_ fixation and nitrate assimilation in cyanobacteria and algae**.

**Species name**	**Organism type**	**Aquatic habitat**	**Mo requirements by N source**	**Mo:C (μmol mol^−1^)**	**References**
*Nostoc/Anabaena* sp.	Cyanobacterium	Freshwater	N_2_	–	Bortels, 1940
*Chlorella pyrendoisa*	Green alga	Freshwater	NO^−^_3_	–	Walker, 1953; Loneragan and Arnon, 1954
*Anabaena cylindrica*	Cyanobacterium	Freshwater	N_2_ > NO^−^_3_ > NH^+^_4_	–	Fogg and Wolfe, 1954; Wolfe, 1954; Fay and Vasconcelos, 1974; Jacob and Lind, 1977; Attridge and Rowell, 1997
*Anabaena oscillarioides*	Cyanobacterium	Freshwater	N_2_	0.1–3^*a*^	ter Steeg et al., 1986
*Scenedesmus obliquus*	Green alga	Freshwater	NO^−^_3_ > NH^+^_4_ ≅ urea	–	Arnon et al., 1955; Ichioka and Arnon, 1955
*Scenedesmus acutus*	Green alga	Freshwater	NO^−^_3_	–	Glass, 2011
*Navicula pelliculosa*	Diatom	Freshwater	NO^−^_3_	–	Wallen and Cartier, 1975
*Chlamydomonas reinhardtii*	Green alga	Freshwater	NO^−^_3_	–	Wallen and Cartier, 1975
*Anacystis nidulans*^*b*^	Cyanobacterium	Freshwater	NO^−^_3_ > NO^−^_2_ > NH^+^_4_	–	Peschek, 1979
*Anabaena variabilis* ATCC 29413	Cyanobacterium	Freshwater	N_2_	–	Attridge and Rowell, 1997; Zerkle et al., 2006
*Nostoc sp.* PCC 7120	Cyanobacterium	Freshwater	N_2_ > NO^−^_3_	0.2–132	Glass et al., 2010
*Nostoc sp.* CCMP 2511	Cyanobacterium	Coastal	N_2_ > NO^−^_3_	0.5–5.8	Glass et al., 2010
*Aphanizomenon sp.*	Cyanobacterium	Coastal	N_2_	0.8–2.0	Walve and Larsson, 2007
*Nodularia spumigena*	Cyanobacterium	Coastal	N_2_	0.8–2.0	Walve and Larsson, 2007
*Trichodesmium erythraeum* strain IMS101	Cyanobacterium	Marine	N_2_	1.0–6.6	Tuit et al., 2004
*Trichodesmium* (field samples from North Atlantic)	Cyanobacterium	Marine	N_2_	9–54	Tuit et al., 2004; Nuester et al., 2012
*Crocosphaera watsonii* strain WH8501	Cyanobacterium	Marine	N_2_	0.6–0.9	Tuit et al., 2004

a Calculated by assuming that 45% of dry biomass weight is carbon.

b Renamed Synechococcus elongatus PCC 7942.

### Nitrogen fixation

The first reports that Mo was a bioessential element for N_2_ fixation were published by the German microbiologist Hermann Bortels on the soil bacterium *Azotobacter chroococum* (Bortels, 1930) and the aquatic heterocystous cyanobacterium *Nostoc*/*Anabaena* (Bortels, 1940). Since Bortels' early studies, *Nostoc*/*Anabaena* and *Azotobacter* strains have been model organisms for the study of Mo requirements for N_2_ fixation. Subsequent studies showed that *Anabaena* had optimal growth at dissolved Mo concentrations in the range of 50–2000 nM (Wolfe, 1954; Jacob and Lind, 1977; ter Steeg et al., 1986; Attridge and Rowell, 1997) and that Mo limitation of N_2_ fixation occurred at 1–5 nM Mo (Fay and Vasconcelos, 1974; Attridge and Rowell, 1997; Zerkle et al., 2006; Glass et al., 2010). The onset of Mo limitation requires several transfers to ensue, likely due to the expression of high-affinity ModABC MoO^2−^_4_ uptake systems, which are widely distributed in bacteria and archaea (Self et al., 2001; Zhang and Gladyshev, 2008) and have been characterized in *Nostoc/Anabaena* (Thiel et al., 2002; Zahalak et al., 2004).

All of the studies mentioned above were performed on freshwater cyanobacteria (*Nostoc/Anabaena*). To our knowledge, no previous studies have explored the effect of varying Mo concentration on marine microbes, although one previous study looked at such an effect on coastal heterocystous cyanobacteria (Glass et al., 2010). In recent years there have been a number of reports of intracellular Mo abundances in marine cyanobacteria that enable us to compare approximate Mo requirements between freshwater, coastal, and marine cyanobacteria (Table 1). When grown at Mo concentrations typical of freshwaters (<20 nM), freshwater heterocystous cyanobacteria have Mo:C ratios of 0.1–3 μmol mol^−1^ (ter Steeg et al., 1986; Glass et al., 2010). When grown at higher Mo concentrations, they can accumulate Mo up to extremely high levels (>100 μmol mol^−1^; Glass et al., 2010). Cultured coastal heterocystous cyanobacteria have Mo:C ratios of 2–4 μmol mol^−1^ when grown at seawater Mo concentrations, similar to the natural range observed in the heterocystous cyanobacteria *Aphanizomenon* sp. and *Nodularia spumigena* sampled from the Baltic Sea (Walve and Larsson, 2007). Interestingly, marine non-heterocystous cyanobacteria *Trichodesmium erythraeum* strain IMS101 and *Crocosphaera watsonii* strain WH8501 have similar Mo quotas to freshwater and coastal cyanobacteria when cultivated in the laboratory (0.6–6.6 μmol mol^−1^ Mo:C), but *Trichodesmium* sp. sampled in the North Atlantic have much higher intracellular Mo (9–54 μmol mol^−1^ Mo:C; Tuit et al., 2004; Nuester et al., 2012), suggesting that they may have higher Mo requirements than other cyanobacteria and/or that they have a mechanism for Mo storage.

Organisms with alternative nitrogenases (containing vanadium (V) or iron (Fe) in place of Mo) express them instead of Mo nitrogenase under Mo limitation (Robson et al., 1986). Such alternative nitrogenases are rare in heterocystous cyanobacteria but are more common in soil bacteria such as *Azotobacter* (Betancourt et al., 2008), likely because the average V concentration in soils is ~100-fold higher than Mo (Gupta, 1997). Alternative nitrogenases are less efficient at N_2_ fixation than Mo nitrogenases and thus Mo nitrogenases are expressed when sufficient Mo is available (Eady, 1996). Recently, it was discovered that *Azotobacter vinelandii* produces organic compounds under Mo and V limitation that form strong complexes with Mo and V and are available for cellular uptake (Liermann et al., 2005; Bellenger et al., 2008, 2011). Such “molybdophores” are one of several mechanisms that N_2_ fixers possess for combatting Mo limitation, with other examples including high affinity Mo uptake systems and Mo storage proteins, including MoSto (Pienkos and Brill, 1981; Fenske et al., 2005) and Mop (Hinton and Merritt, 1986; Schüttelkopf et al., 2002; Pau, 2004).

### Nitrate assimilation

Mo was first reported to be essential for NO^−^_3_ assimilation in the fungus *Aspergillus niger* (Steinberg, 1937), which required additional Mo when cultivated on media containing NO^−^_3_ than NH^+^_4_. Follow-up studies showed that this higher Mo demand for growth on NO^−^_3_ than NH^+^_4_ was also present in green algae *Chlorella pyrendoisa* (Walker, 1953) and *Scenedesmus obliquus* (Arnon et al., 1955; Ichioka and Arnon, 1955) and the cyanobacterium *Anacystis nidulans* (later renamed *Synechococcus elongatus* PCC 7942; Peschek, 1979). We found that the green alga *Scenedesmus acutus* grown in chemostats contained 60% higher chlorophyll *a*, 3000% higher nitrate reductase activity and 80% higher cellular Mo when grown on high concentrations of Mo (90 μM) than under Mo limitation (1 nM; Glass, 2011). Studies of the purified assimilatory nitrate reductase verified that the enzyme requires Mo for activity (Nicholas and Nason, 1954; Nicholas et al., 1954; Vega et al., 1971) and contains one molybdopterin cofactor per active site (Solomonson et al., 1986).

Different organisms vary in how much Mo they require for NO^−^_3_ assimilation. For instance, the freshwater diatom *Navicula pelliculosa* is more susceptible to Mo limitation of photosynthesis and NO^−^_3_ uptake than the freshwater green alga *Chlamydomonas reinhardtii* (Wallen and Cartier, 1975). This may be due to the presence of a high-affinity eukaryotic MoO^2−^_4_ uptake system (MOT1) in *C. reinhardtii* that is absent from diatoms (Tejada-Jimenez et al., 2007). The spotty distribution of MOT1 in eukaryotes was confirmed by a recent bioinformatic survey (Zhang and Gladyshev, 2010). It is possible that eukaryotes containing MOT1 or other unknown high-affinity Mo transporters can cope with Mo limitation for longer time periods than other organisms that lack such transporters.

### Comparison of Mo requirements

Models and experiments have demonstrated that N_2_ fixation requires more Mo than NO^−^_3_ assimilation, while other more chemically-reduced forms of N, such as NH^+^_4_, do not require Mo for assimilation. This is because the specific reaction rate of nitrate reductase (mol N assimilation per mol Mo s^−1^) is 2–3 orders of magnitude higher than nitrogenase (Sprent and Raven, 1985). Based on this difference in activity, it has been calculated that a N_2_-fixing cell requires ~125× more Mo per cell than a NO^−^_3_-grown cell (Sprent and Raven, 1985; Raven, 1988). The greater need for Mo during N_2_ fixation vs. NO^−^_3_ assimilation was experimentally demonstrated for both cyanobacteria and legumes containing symbiotic N_2_ fixers in their root nodules (Anderson and Spencer, 1949, 1950; Fogg and Wolfe, 1954; Wolfe, 1954). In accordance with this higher need, cellular Mo quotas are also higher when cyanobacteria are grown on N_2_ than NO^−^_3_ (Glass et al., 2010).

## Field studies

Dissolved Mo concentrations have been measured in a large number of globally distributed freshwater lakes, and range from <0.1–13 nM (Table 2). Mo concentrations increase with salinity, and therefore saline lakes often contain much higher concentrations of Mo than freshwater lakes (Marino et al., 1990; Johannesson et al., 2000). In this section we review previous studies on Mo limitation in freshwater lakes, with particular emphasis on Castle Lake in northern California, USA. We summarize new data for Castle Lake, including results of experiments exploring the influence of Mo and NO^−^_3_ additions on the activity of enzymes involved in NO^−^_3_ assimilation, and dissolved Mo depth profiles. In later sections we discuss the possibility of Mo limitation of N assimilation in marine and soil environments. Mo has been clearly shown to limit N_2_ fixation in temperate and tropical soil ecosystems, whereas the evidence is more ambiguous for marine environments.

**Table 2:** **Dissolved Mo concentrations for Castle Lake and other freshwater lakes around the world**.

**Lake name and location (number of lakes in study)**	**Dissolved Mo (nM)**	**References**
Castle Lake, California, USA	2–4	Bachmann and Goldman, 1964; Glass, 2011
Clear Lake, Colorado, USA	1–4	Elser and Glass, unpublished data
Esthwaite Water, England	0.1–2.6	Achterberg et al., 1997
Linsley Pond, Connecticut, USA	0.4–2.7	Cowgill, 1977
New Zealand lakes (3)	<0.7	Goldman, 1964
Alaska lakes (3)	≤0.6	Goldman, 1964
Mirror Lake, New Hampshire, USA	0.1–0.3	Cole et al., 1986
Japan lakes (13)	0.5–13	Sugawara et al., 1961
Lake Greifen, Switzerland	3–5	Magyar et al., 1993
Lake Insjön, Sweden	6.4	Lithner et al., 2000
Lake Lundsjön, Sweden	0.8	Lithner et al., 2000
Eastern Canadian lakes (4)	0.1–3.4	Chappaz et al., 2008
Hall Lake, Washington, USA	1–2	Balistrieri et al., 1994
Sierra Nevada lakes (170), California, USA	0.03–10	Bradford et al., 1968
Northern Germany lakes (8)	0.5–10	Groth, 1971
Amazonas, Brazil lakes (3)	4–8	Groth, 1971

### Mo limitation in castle lake and other freshwaters

#### Previous studies

**Figure 1:**
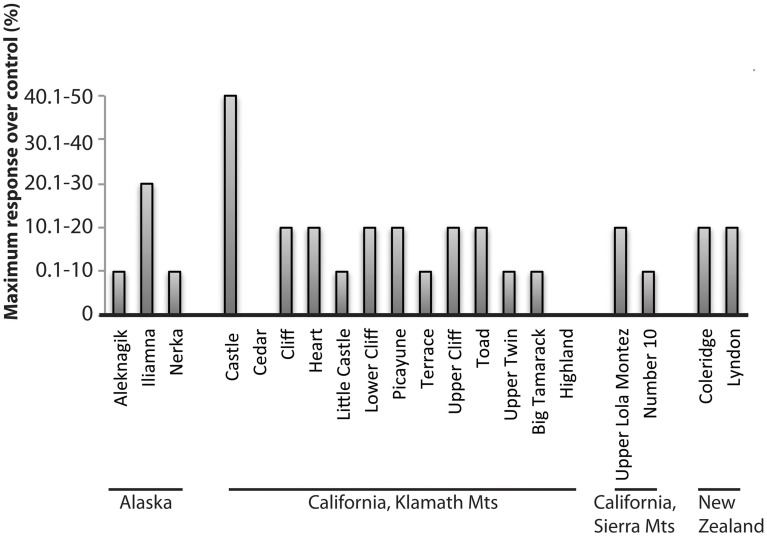
**Maximum response of bottle incubations after addition of 40 nM Mo in 20 lakes, in units of percent increase over control.** Compiled from Goldman (1964).

Early studies tested the response of Mo additions to oligotrophic lakes in Alaska, California, and New Zealand (Goldman, 1964). Mo additions to bottle incubations were performed for 3 lakes in Alaska, 13 lakes in the Klamath Mountains of California, 2 lakes in the Sierra Nevada Mountains of California and 2 lakes in New Zealand (Figure 1). Mo concentrations in Lake Aleknagik (Alaska) and Lakes Coleridge and Lyndon (New Zealand) were <0.7 nM (Goldman, 1964) and lakes in the Sierra Nevada averaged 4 nM (Bradford et al., 1968). Response to Mo additions was measured by comparing photosynthetic H^14^CO^−^_3_ uptake between control bottles and bottles amended with 40 nM Mo. The majority of lakes showed a significant increase in ^14^C uptake after Mo addition: 0.1–10% increase over controls (7 lakes), 10.1–20% increase over controls (9 lakes), 20.1–30% increase over controls (1 lake) and 40.1–50% increase over controls (1 lake; Figure 1). Only 2 lakes of the 20 tested did not respond to Mo additions.

The lake with the most positive response to Mo addition of the 20 tested was Castle Lake, a small (0.2 km^2^) meso-oligotrophic, subalpine lake in the Klamath Mountains of northern California with low dissolved Mo (2–4 nM). These low Mo concentrations are likely a result of the ultramafic deposits underlying the lake's watershed, which contain very low concentrations of Mo (Kuroda and Sandell, 1954; Glass, 2011; Glass, J. B., Chappaz, A., Eustis, B., Heyvaert, A. C., Waetjen, D., Hartnett, H. E., and Anbar, A. D., Molybdenum geochemistry in Castle Lake, California, in review, *Geochim. Cosmochim. Acta*). Addition of 1 μM Mo increased the rate of photosynthesis in lake surface phytoplankton communities in the summer, fall, and winter months (Goldman, 1960). Lower concentrations of Mo stimulated primary productivity in January than in June or October, suggesting that planktonic communities were more Mo-limited during the winter than the summer. This explanation is reasonable given that Castle Lake water sampled from under the ice had lower dissolved Mo concentrations (0.1–0.9 nM; Glass, 2011; Glass et al., in review) than surface lake water sampled in the mid-summer 2008 and 2009 (2.4–2.9 nM; Figure 3A; see below). Similarly, pronounced winter depletions of Mo have been measured in Lake Donk, Belgium (Dumont, 1972) and Linsley Pond, Connecticut (Cowgill, 1977). For more on the geochemical cycling of Mo in Castle Lake, see Glass et al., (in review).

Two experimental whole-lake additions of Mo were performed in Castle Lake in the 1960s. In July 1963 and again in June 1969, 6.3 kg of Mo was added as solid-phase sodium molybdate (Na_2_MoO_4_) to the lake's epilimnion to stimulate primary productivity (Goldman, 1966, 1972). These Mo additions increased dissolved Mo in the epilimnion from ~2–5 nM to 50–80 nM. Following the 1963 experimental addition of Mo to the lake, primary productivity in the epilimnion increased 40% over the previous year (Goldman, 1966). The golden alga *Dinobryon sertularia* exhibited the largest increase in cell numbers (Goldman, 1972; Jassby and Goldman, 1974). Trout, cladoceran, and copepod yields also increased.

Subsequent studies showed that Mo limitation of primary productivity in Castle Lake was likely a result of insufficient Mo for maximal rates of NO^−^_3_ assimilation. Additions of 50 nM Mo to bottle incubations of Castle Lake water led to increased NO^−^_3_ uptake (up to 60%), but only when NO^−^_3_-N concentrations were >1 μM and NH^+^_4_-N was <0.1 μM (Axler et al., 1980). Regeneration of NH^+^_4_ by zooplankton excretion and microbial mineralization are important N sources for the Castle Lake phytoplankton community (Axler et al., 1981; Zehr et al., 1985). In the epilimnion, NH^+^_4_ assimilation rates were significantly higher than those of NO^−^_3_ throughout the summer (Axler et al., 1981, 1982) and mid to late-summer regeneration of NH^+^_4_ contributed more than 50% of the total N assimilated (Axler et al., 1981). Small amounts of NH^+^_4_-N (0.3–0.4 μM) were shown to inhibit NO^−^_3_ assimilation by more than 75% in the Castle Lake epilimnion (Priscu et al., 1985), whereas higher NH^+^_4_ concentrations were required for inhibition at greater depths. At 20 m, ~3 μM NH^+^_4_-N was required to significantly inhibit NO^−^_3_ assimilation, whereas at 25 m, enrichment with 5.4 μM NH^+^_4_-N resulted in minimal inhibition (Priscu et al., 1985). From 1976–2010, mean NH^+^_4_-N during the ice-free growing season (June through mid-September) was 0.3 ± 0.2 (mean±1 s.d.) μM in the epilimnion at 3 m, 0.3 ± 0.2μM in the upper hypolimnion at 15 m and 1.0 ± 0.9μM in the mid-hypolimnion at 25 m (CLEREP, 2011). Therefore, the epilimnetic phytoplankton community is probably more susceptible to NH^+^_4_ suppression of NO^−^_3_ assimilation than the hypolimnetic community, where mean NH^+^_4_ levels are typically below the inhibition threshold (Axler et al., 1980; Axler and Goldman, 1981; Priscu et al., 1985). Thus, it is reasonable to expect that Mo requirements increase with depth in Castle Lake if NO^−^_3_ is a more important N source in the hypolimnion than in the epilimnion.

### Current study: enzyme bioassays

**Figure 2:**
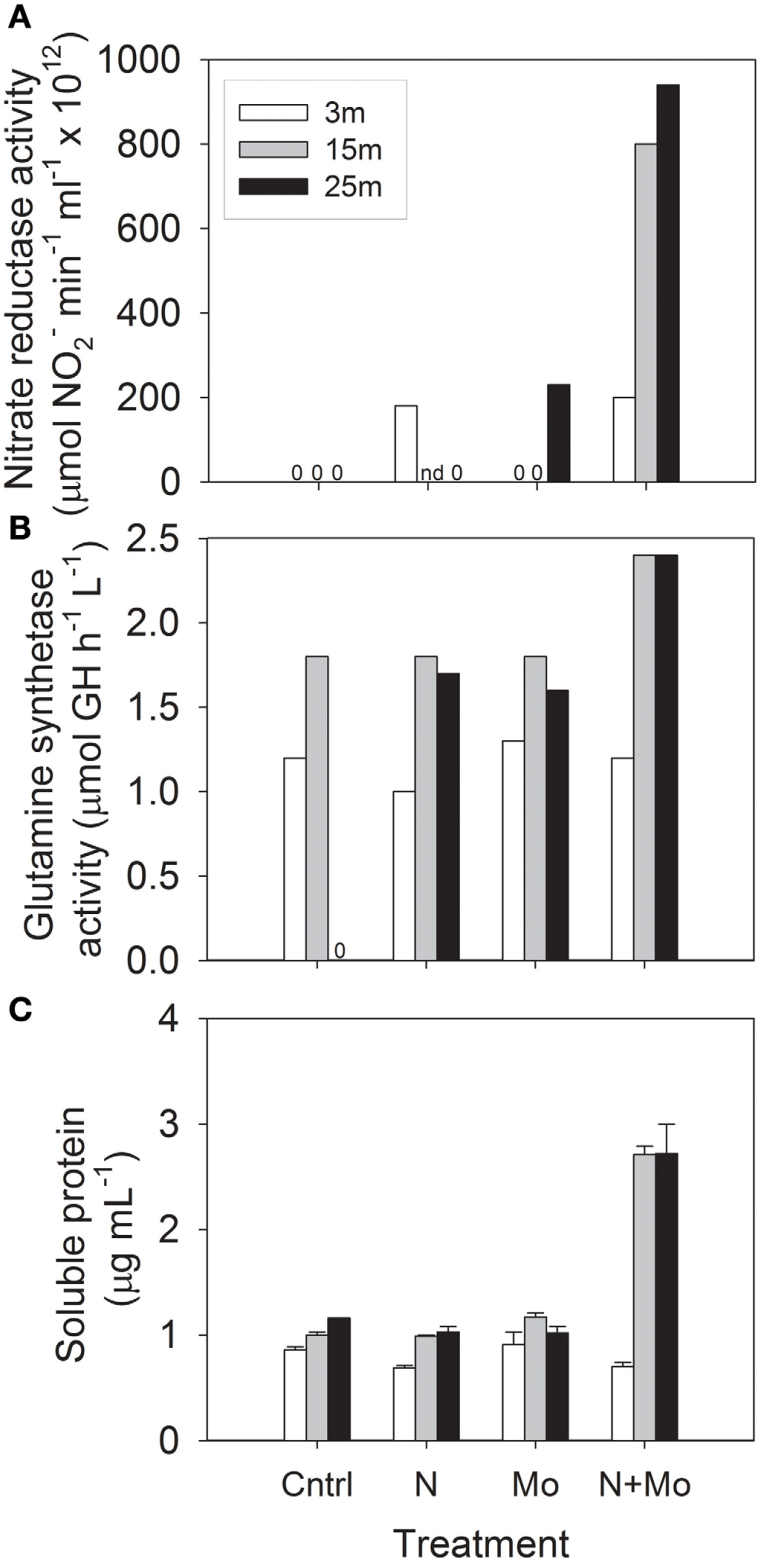
**Results of bioassay experiments at three depths in Castle Lake in July 2008 for control (Cntrl), +nitrate (N), +molybdenum (Mo), and +nitrate +molybdenum (N + Mo) treatments: (A) nitrate reductase activity; (B) glutamine synthetase activity; (C) soluble protein content (“0” stands for undetectable activity and “nd” stands for “no data” “GH” stands for γ-glutamyl hydroxamate).** After Glass (2011).

Building on previous isotopic tracer (^15^N, ^13^N, and ^14^C) uptake experiments in Castle Lake (Goldman, 1960; Axler et al., 1980, 1982; Axler and Goldman, 1981; Axler and Reuter, 1996), we investigated the effect of Mo availability on the enzyme activity of key N assimilation proteins and total protein abundances at three depths in Castle Lake. In July 2008, we assayed the activity of the first and third enzymes involved in NO^−^_3_ assimilation (the Mo-containing eukaryotic nitrate reductase and the non-metal-containing enzyme glutamine synthetase) and total protein content after the addition of 100 μM NO^−^_3_ as NaNO_3_ and 100 nM Mo as Na_2_MoO_4_ to bottle experiments (background NO^−^_3_-N concentrations at the start of the experiment were 0.1 μM at 3 m, 0.2 μM at 15 m, and 1.6 μM at 25 m; background Mo was 2–4 nM; see Glass, 2011 for experimental details). These experiments showed a consistent positive response at the lower two depths (15 and 25 m), but only in the +N +Mo treatments (Figure 2). This hypolimnetic response occurred for all three measurements: nitrate reductase activity (Figure 2A), glutamine synthetase activity (Figure 2B), and soluble protein content (Figure 2C), suggesting that supplemental Mo enabled added NO^−^_3_ to be assimilated into protein. Ambient NH^+^_4_-N concentrations were 0.2 μM at 3 m and ≤0.02 μM at 15 and 25 m. The lack of response to +N +Mo treatments at 3 m suggests that 0.2 μM NH^+^_4_-N, slightly lower than the inhibition threshold of 0.3–0.4 μM NH^+^_4_-N reported by Priscu et al. (1985), was inhibiting NO^−^_3_ uptake. The strong response to +N +Mo additions at 15 and 25 m suggests that hypolimnetic microbial communities were Mo-NO^−^_3_ co-limited; that is, addition of only one nutrient did not stimulate enzyme activity and protein synthesis because the other nutrient was limiting—only the simultaneous addition of both nutrients produced a positive response. This finding is in accordance with scarce NH^+^_4_ (≤0.02 μM NH^+^_4_-N) in the hypolimnion, reducing the possibility of NH^+^_4_ inhibition.

### Current study: Mo depth profiles

**Figure 3:**
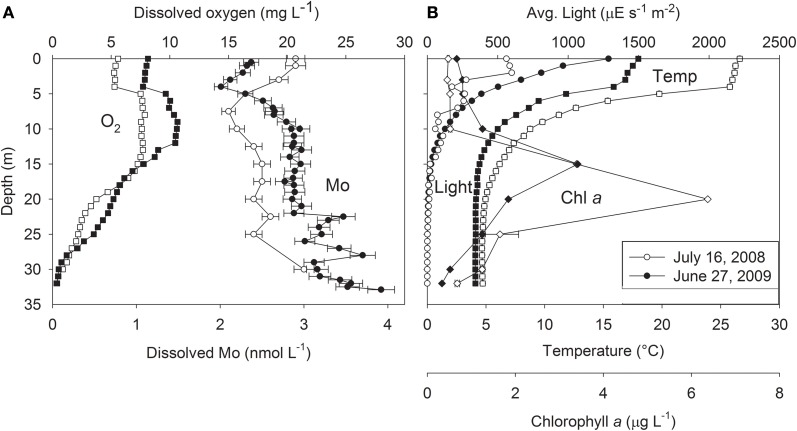
**(A)** Dissolved oxygen (O_2_; squares) and dissolved molybdenum (Mo; circles), and **(B)** average light (circles), temperature (squares), and chlorophyll *a* concentrations (Chl *a*; diamonds) with depth in Castle Lake, from water column samples collected on 16 July 2008 (white symbols) and 27 June 2009 (black symbols). After Glass (2011).

We extended previous studies of Castle Lake Mo bioavailability (Bachmann and Goldman, 1964; Goldman, 1966) by obtaining high-resolution dissolved Mo depth profiles from the center of Castle Lake on 16 July 2008 and 27 June 2009 (see Glass, 2011 for details). The dissolved Mo content of the Castle Lake water column ranged from 2 to 4 nM (Figure 3), similar to values reported from the 1960s using different analytical methods. Surface water Mo concentrations were 2.9 and 2.4 nM in 2008 and 2009, respectively. Both years, Mo concentration minima occurred near the thermocline, where Mo was lower by 0.5–0.8 nM than in shallower and deeper waters. The concentration minimum was offset ~5 m deeper in 2008 than 2009. The Mo minimum was located at a shallower depth (5–10 m) than the deep chlorophyll *a* maximum (15–20 m), whereas Mo minima and dissolved O_2_ maxima occurred at similar depths (Figures 3A,B). Dissolved Mo was relatively constant down to about 20 m, and then increased toward the lake bottom, suggesting that sediments are a source of Mo to the lake in the mid-summer (Glass et al., in review; Figure 3A).

The finding of Mo minima in the Castle Lake epilimnion suggests that dissolved Mo is being taken up biologically in excess of that supplied by watershed and sediment inputs. The concurrence of dissolved Mo minima and dissolved O_2_ maxima near the thermocline may provide clues as to which Castle Lake microbial community is responsible for the prominent Mo drawdown between 5 and 10 m depth. Positive heterograde O_2_ profiles like those of Castle Lake, exhibiting an O_2_ maximum at the thermocline, can arise from high phytoplankton photosynthesis and/or diffusion of O_2_ (produced by submerged macrophytes, periphyton, or phytoplankton) from the littoral zone into the thermocline (Wetzel, 2001). The littoral zone of Castle Lake is an extensive shallow (3–5 m) platform with a well-studied benthic periphyton community that includes an expansive epipelic (i.e., living freely on sediment surfaces) plain comprised of a mixed community of diatoms, chlorophytes, and non-heterocystous cyanobacteria, and a fringing epilithic (i.e., growing on a rock surface) community dominated by N_2_-fixing heterocystous cyanobacteria (Sanders, 1976; Loeb and Reuter, 1981; Reuter et al., 1985; Reuter and Axler, 1988, 1992; Axler and Reuter, 1996), that have high Mo requirements (see above). Even when epilimnetic water is severely depleted in NO^−^_3_, the overlying epipelic periphyton can still obtain a significant amount of their N-nutrition by fixing N_2_ in the dark when they are not producing O_2_ (Bergman et al., 1997; Berman-Frank et al., 2003) or by assimilating interstitial porewater NO^−^_3_ that diffuses upward from the sediments (Reuter et al., 1985; Reuter and Axler, 1992). Therefore, this microbial community represents a potentially important sink for available Mo. Neither heterocystous cyanobacteria, nor N_2_-fixation, have been found in the planktonic communities of the lake over the many years of intensive monitoring (Reuter et al., 1985; Goldman, unpublished). This fact, together with the extremely low rates of planktonic NO^−^_3_ assimilation rates previously measured in the epilimnion (Gersberg et al., 1978; Axler and Goldman, 1981; Axler et al., 1982), suggests that the Mo minimum at the thermocline likely represents demand by littoral periphyton communities that fix N_2_ and also assimilate NO^−^_3_. This raises the question of whether these periphyton communities are limited for Mo. One previous experiment provides support for this hypothesis: N_2_-fixation increased 210% when 50 nM Mo was added to a lake containing <10 nM Mo in the Great Salt Basin during a cyanobacterium (*Aphanizomenon flos-aquae*) bloom (Wurtsbaugh, 1988). However, Mo requirements for periphyton microbial communities remain to be determined.

### Marine and saline environments

A long-standing question in oceanography is the cause for widespread N limitation in marine environments (Vitousek and Howarth, 1991). Mo was long dismissed as a potential limiter of marine N assimilation due to its high seawater concentration and conservative behavior with depth (~107 nM; Collier, 1985). Robert Howarth and colleagues challenged that assumption by suggesting that high levels of seawater sulfate may compete with Mo uptake (Howarth and Cole, 1985; Howarth et al., 1988). Sulfate (SO^2−^_4_) and molybdate (MoO^2−^_4_, the dominant species of Mo in oxic waters) have very similar size and stereochemistry, but sulfate (SO^2−^_4_; 28 mM) is ~10^5^-fold more abundant than molybdate (MoO^2^_4_; 100 nM) in seawater. Amendment experiments in the Baltic Sea (with ambient Mo of 20 nM and sulfate of 4.8 mM, a S:Mo ratio of 240,000) showed that additions of 100 nM Mo (S:Mo ratio of 40,000) led to 50% higher N_2_ fixation rates than the control, whereas additions of 5 mM sulfate (S:Mo ratio of 490,000; roughly 2-fold higher than seawater) led to N_2_ fixation rates 25% lower than the control (Howarth and Cole, 1985). However, N_2_ fixation rates were low in this experiment and the authors warned against extrapolation to marine systems. In follow-up studies with pure cultures and freshwater systems, Mo uptake was inhibited at S:Mo ratios 50–100-fold lower than those present in seawater (Cole et al., 1986, 1993) and addition of sulfate to seawater levels greatly reduced N_2_ fixation rates (Marino et al., 2003). Also consistent with this hypothesis, the S:Mo ratio in 13 saline lakes in Alberta, Canada was negatively correlated with the abundance of N_2_-fixing cyanobacteria (Marino et al., 1990).

While these studies lent support to the S/Mo inhibition hypothesis, others challenged it. *In situ* bioassays performed off the North Carolina coast showed that addition of 0.1–5 μM Mo resulted in no significant change in N_2_ fixation rates (Paerl et al., 1987; Paulsen et al., 1991). Similarly, mesocosm studies with water sampled from Narragansett Bay, Rhode Island produced no significant change in heterocyst abundance (a proxy for N_2_-fixing cell abundance) when Mo was increased from ambient levels (98 nM) to 1.23 μM under highly N-limited conditions (Marino et al., 2003). Additions of Mo to the Great Salt Lake, which lowered S:Mo ratios from 299,00:1 to 83,000:1, did not significantly stimulate N_2_ fixation (Wurtsbaugh, 1988). Similarly, Mo additions to 8 of the 13 saline lakes studied by Marino et al. (1990) had no significant effect on phytoplankton biomass (Evans and Prepas, 1997). Finally, Mo uptake by *Azotobacter vinelandii* was not inhibited at S:Mo ratios up to eight times that of seawater (Paulsen et al., 1991). Overall, it is still unclear whether high SO^2−^_4_ inhibits Mo uptake in marine environments, as this question has never been tested in open ocean conditions. However, it seems that marine microbes are adapted to discriminate between SO^2−^_4_ and MoO^2−^_4_ in a manner that freshwater microbes are not.

### Soil habitats

Since this paper is focused on Mo limitation of aquatic environments, we will touch only briefly on Mo limitation of soil ecosystems. For a much more thorough review of Mo as it relates to soils, crops, and livestock, see Gupta (1997). Studies on Mo deficiencies in soils date back to the 1930s, when Arnon and Stout (1939) first showed that Mo was an essential micronutrient for higher plants. Nitrate was found to accumulate in plant leaf tissues when Mo was omitted from the nutrient medium, presumably because it could not be assimilated into organic matter (Hewitt and Jones, 1947). Mo limitation is especially prevalent on serpentine soils which contain less Mo than other soil types (Walker, 1948). The importance of Mo for symbiotic N_2_ fixation in alders containing N_2_-fixing bacteria in their root nodules was first shown by Becking (1961a,b). Goldman (1960, 1972) proposed that alders growing along the shore of Castle Lake may scrub soluble Mo from soil porewaters, thereby competing with the aquatic ecosystem for scarce Mo. More recently, N_2_ fixation has been found to be limited by Mo availability in both temperate and tropical soils (Silvester, 1989; Barron et al., 2009; Wurzburger et al., 2012). This effect may be exacerbated by rising atmospheric CO_2_: Hungate et al. (2004) found that rising atmospheric CO_2_ led to increased legume N_2_ fixation during the first year of treatment of an oak woodland, but this response declined and disappeared by the third year and N_2_ fixation was depressed in the fifth, sixth, and seventh years. This effect was best explained by declines in foliar Mo concentrations with increased atmospheric CO_2_, suggesting that the bioavailability of Mo in soils declined, likely either by decreased pH or accumulation of organic matter leading to increased sorption of Mo onto particles. Therefore, Mo limitation of N assimilation may limit the ability of both terrestrial environments (and possibly aquatic ones as well) to serve as sinks for anthropogenic CO_2_ emissions.

## Conclusions and future directions

Research on Mo requirements for microbial N assimilation dates back to the 1930s and has recently been rejuvenated by the important finding that N_2_ fixation may be limited by decreased Mo bioavailability under increased atmospheric CO_2_ (see above). Studies of pure cultures of cyanobacteria, algae, and soil bacteria have shown that Mo requirements are highest when organisms are fixing N_2_, lower but still significant when grown on NO^−^_3_, and negligible when grown on NH^+^_4_. Furthermore, there are differences in Mo requirements among species for a given N source, likely due to the presence or absence of high-affinity Mo uptake systems and/or other Mo scavenging mechanisms. *In situ* experiments with natural populations of microbes in lakes have shown that Mo limitation of primary productivity may be widespread, particularly in freshwaters underlain by geologic deposits containing scarce Mo, such as Castle Lake in northern California. Data presented here support the theory, originally put forth in previous studies (Goldman, 1960; Axler et al., 1980; Axler and Goldman, 1981), that Mo availability can limit NO^−^_3_ assimilation in Castle Lake when NH^+^_4_ is scarce, and show that this limitation manifests itself in suppressed enzyme activity and protein content. Furthermore, we found circumstantial evidence in Castle Lake Mo and O_2_ depth profiles that N_2_ fixation by periphyton communities leads to appreciable dissolved Mo drawdown and thus may also be Mo-limited, an idea that requires future testing. Below we discuss up-and-coming research topics related to Mo-N co-limitation: the bioavailability of different redox states of Mo, the use of Mo isotope systematics to elucidate microbial Mo uptake mechanisms, the possibility of Mo limitation of N_2_ fixation in the marine environment, and the co-evolution of microbial metabolisms with changing availability of Mo in the ocean through time.

### Bioavailability of Mo in different oxidation states

While the major Mo species in oxic waters is Mo(VI)O^2−^_4_, Mo(V) was found to comprise up to 15% of total Mo concentrations in estuarine waters and likely a significant portion of total Mo in many aquatic systems (Wang et al., 2009). It has been proposed that reduced Mo(V) is more bioavailable than oxidized Mo(VI) as MoO^2−^_4_ because Mo^5+^ is the redox state of Mo in some enzymes (see references in Wang et al., 2009) and does not compete with SO^2−^_4_ during uptake (Howarth and Cole, 1985; Howarth et al., 1988). Recent experiments support this hypothesis: in California lakes of varying trophic status (Lake Tahoe, Walker Lake, and Clear Lake), N_2_ fixation rates and chlorophyll concentrations were positively correlated with Mo(V) concentrations, which increased with trophic status from Lake Tahoe to Lake Walker to Clear Lake (Romero et al., 2011). Under reducing conditions often present in sediments and bottom waters, a higher proportion of Mo will be reduced (Wang et al., 2011) or converted to particle-reactive thiomolybdate (Mo(VI)O_x_S^2−^_4−x_) if H_2_S is present in relatively high concentrations (Helz et al., 1996, 2011; Erickson and Helz, 2000; Vorlicek and Helz, 2002; Vorlicek et al., 2004). Very little is known about the bioavailability of thiomolybdate (and other chemical species of Mo besides Mo(VI)O^2−^_4_); this is a topic that deserves more attention.

### Mo isotopic fractionation

Recently, studies revealed that *Nostoc/Anabaena* and *Azotobacter* fractionate Mo isotopes with distinct fractionation factors when fixing N_2_ (Wasylenki et al., 2007; Zerkle et al., 2011). This fractionation likely occurs during uptake by the ModABC system (see above). Although this research is still in its early phases, future studies may benefit from using Mo isotopes to discern the intricacies of Mo biological pools in microbial species.

### Mo limitation in the marine environment?

Considering the importance of N_2_ fixation in the marine environment (Sohm et al., 2011 and references therein), surprisingly few studies have investigated Mo requirements for N_2_-fixing marine microbes compared to the greater number of studies on freshwater microbes. The reason for this gap is likely due to the assumption that dissolved Mo is too abundant in seawater to be a limiting micronutrient (see above). However, non-conservative behavior of Mo in coastal regions has been observed; dissolved Mo drops as low as 30 nM in the Wadden Sea (Dellwig et al., 2007). Although this concentration is still higher than has been shown for Mo requirements in freshwater and (one strain of) coastal cyanobacteria, it is possible that other coastal and open ocean strains require higher Mo, a hypothesis that requires future testing. Furthermore, it is quite possible that N_2_ fixation in marine sediments (e.g., Dekas et al., 2009) is Mo-limited, as dissolved Mo in marine sediment porewaters may be reduced or converted to particle-reactive thiomolybdate (see above), leaving low levels of bioavailable Mo for microbial N_2_ fixation. Alternatively, microbes may have evolved yet-unknown mechanisms to access Mo in sulfidic sediment porewaters. Finally, the S/Mo inhibition hypothesis still needs to be tested in open ocean conditions. If no inhibition is identified, a thorough study of the differences in MoO^2−^_4_/SO^2−^_4_ uptake pathways is needed, as that would imply that marine microbial uptake systems have more specificity for Mo than those of freshwater microbes.

### Ancient ocean Mo chemistry and evolution

While Mo is the most abundant transition metal in the modern ocean, it was likely much scarcer in seawater earlier in earth history and may have been an important limiting micronutrient of marine primary productivity. Before the Great Oxidation Event (GOE) ~2.4 billion years ago, the riverine flux of Mo to the ocean would have been small due to minimal oxidative weathering of sulfides, the major source of Mo on the continents (Scott et al., 2008). Nevertheless, Mo proteins seem to have very ancient roots and likely evolved in a low-Mo ocean (Raymond et al., 2004; Boyd et al., 2011; Schoepp-Cothenet et al., 2012). After the GOE up until ~800 million years ago, the presence of sulfide in the deep ocean (even if present over a relatively small areal extent) may have kept Mo concentrations low enough to limit N_2_ fixation and perhaps stall the evolution of eukaryotic life (Anbar and Knoll, 2002; Glass et al., 2009; Reinhard, C. T., Planavsky, N. J., Robbins, J., Partin, C., Gill, B. C., Lalonde, S. V., Bekker, A., Konhauser, K. O., and Lyons, T. W. Proterozoic ocean redox and evolutionary stasis, in review, *Proc. Natl. Acad. Sci.*). Marine Mo concentrations likely rose to near their present values ~600 million years ago when the ocean became fully oxic (Scott et al., 2008), but during episodes of extreme global warming or mass extinction events that resulted in marine anoxic events, the return of sulfidic conditions to widespread areas of the deep sea would have caused seawater Mo depletion (Algeo, 2004). While it is not possible to directly extrapolate from the physiology of modern organisms to those of their ancestors, studying the Mo (and other trace metal) requirements of modern microbes living in environments with scarce Mo can provide us with a possible analogue to ancient ecosystems.

### Conflict of interest statement

The authors declare that the research was conducted in the absence of any commercial or financial relationships that could be construed as a potential conflict of interest.
